# Bioprocessing and integration of a high flux screening systematic platform based on isothermal amplification for the detection on 8 common pathogens

**DOI:** 10.1007/s00449-020-02423-4

**Published:** 2020-08-29

**Authors:** Huamin Zhong, Hongwei Deng, Ming Li, Huahong Zhong

**Affiliations:** 1 Department of Clinical Laboratory, Guangzhou Women and Children’s Medical Center, Guangzhou Medical University, Guangzhou, 510120 China; 2 Shenzhen Key Laboratory of Ophthalmology, Ocular Trauma Treatment and Stem Cell Differentiation Public Service Platform of Shenzhen, Shenzhen Eye Hospital, Shenzhen, 518040 China

**Keywords:** Nucleic acid, Isothermal amplification, LAMP, High flux screening, Detection

## Abstract

During a large variety of common pathogens, *E. coli*, *P. aeruginosa*, MRSA, MRCNS, *V. parahaemolyticus*, *L. monocytogenes* and *Salmonella* are the leading pathogens responsible for large number of human infections and diseases. In this study, a high flux screening based on nucleic acid isothermal amplification technique has been developed. For the 8 common pathogens, species-specific targets had been selected and analyzed for their unique specificity. After optimization, separate LAMP reaction assays had been bioprocessed and integrated into one systematic detection platform, including 8 strips (PCR tubes) and 96-well plates. Eight standard strains verified for the accuracy. Application of the established high flux screening platform was used for detection for 48 samples in 4 different 96-well plates, with 2 groups of 2 operators using double-blind procedure. The accuracy of 100% was obtained, with the total time consumption as 66–75 min (for 12 samples detection on 8 different pathogens). As concluded, through the bioprocess of the systematic platform based on LAMP technique, it’s been demonstrated to be capable of simultaneous detection of 8 pathogens, with high sensitivity, specificity, rapidity and convenience.

## Introduction

Pathogens mediated infectious diseases in human beings and animals remain a major concern in public health [1]. During a large variety of common pathogens, *E. coli*, *P. aeruginosa*, *S. aureus* (especially methicillin-resistant *Staphylococcus aureus*, MRSA), coagulase-negative staphylococci (MRCNS), *V. parahaemolyticus*, *L. monocytogenes* and *Salmonella* are the leading pathogens responsible for large number of human infections and diseases [2–15]. However, bacterial identification of such pathogens requires up to several days [16]. For example, using “golden standard” culturing takes up 3–7 days depending on the results [17]. The required time length has posed a major concern for clinicians. Early diagnosis of such pathogens is significantly important for further therapeutic treatment and thus substantially influences the prognosis of the infections and diseases [18–20].

In recent years, PCR and Q-PCR have been well studied and documented to be a promising technique for rapid detection and bacterial identification [21–25]. However, regular PCR requires laborious results determination process, such as electrophoresis or hybridization. (The former takes several hours and the latter takes up to 36 h.) For Q-PCR, expensive equipment and reagents are required, which poses an obstacle during its broad application [26, 27]. Since 2000, a novel nucleic acid amplification method has been developed and established, named loop-mediated isothermal amplification (LAMP) [28, 29]. For the past 2 decades, LAMP has been developed for the detection of various microorganisms and other genes [30, 31]. However, the clinical application of LAMP assays, especially platform for high flux screening based on LAMP, has been rarely reported [32–34]. Advantages of LAMP include high sensitivity, specificity, rapidity, simpliness in operation and labor, convenience and expense [35]. However, lack of the bioprocessing and systematic integration of this technique into applicable platforms has been the biggest obstacle during the application of this technique [36–38]. As a consequence, development and application of rapid detection on such pathogens as well as their virulent factors for high flux screening application are of utmost importance and necessity [39–41].

In this study, a high flux screening of pathogens, including *E. coli*, *P. aeruginosa*, MRSA, MRCNS, *V. parahaemolyticus*, *L. monocytogenes* and *Salmonella*, has been developed based on the loop-mediated isothermal amplification methodology.

## Materials and methods

### Bacterial strains

Nine standard strains were used to establish the high flux screening assays in this study, including *E. coli* C600 ATCC25922, *Pseudomonas* aeruginosa: ATCC27853, MRSA 85/2082, MSSA ATCC14458, MRCNS ATCC29887, MSCNS ATCC27844, *Listeria monocytogenes*: ATCC19118, *Salmonella* enterica subsp. enterica: ATCC29629, *Vibrio parahaemolyticus*: ATCC27969. The storage, inoculation, culturing and incubation have been conducted as described previously.

### Design on the systematic integration of detection

Specific targets were selected for different pathogens, with the detailed information as follows. For *E. coli*, rfbE (the specific O-antigen) was selected. For *P. aeruginosa*, oprI was selected. For MRSA, MSSA, MRCNS, MSCNS, mecA, femA and 16SrRNA (specific for the genus of Staphylococci) were selected. For *Salmonella*, invA was selected. For *V. parahaemolyticus*, *tlh* (thermolable haemolysin, considered to be a species-specific marker for *V. parahaemolyticus*) gene was selected. For L. monocytogenes, hlyA was selected. In our previous studies, the primer sets for each of the targets had been separately designed, and optimized for LAMP reaction. Such primer sets were also selected in this study, and additionally, new primer sets for each targets had been designed using Primer Explorer V4 [42–44]. Optimal parameters were selected by each set of primers. Principles for the systematic integration of the high flux screening platform included: firstly, unique temperature is required for the reaction occurred for each primers set. Secondly, unique reaction time is also required for each target. The DNA samples of *E. coli*, *Pseudomonas* aeruginosa, MRSA, MSSA, MRCNS, MSCNS, *L. monocytogenes*, *Salmonella*, *V. parahaemolyticus* were isolated using the DNA extraction kit. DNA quality and concentration had been confirmed with NanoDrop before further detection. LAMP reaction was performed under different temperatures (59 °C to 66 °C), reaction time (0, 15 min, 30 min, 45 min, 60 min, 75 min, 90 min), concentrations of betaine (0.3 M, 0.4 M, 0.5 M, 0.6 M and 0.7 M) and ratios of calcein and Mn2 + (1:20, 1:16, 1:12, 1:8, 1:4, 1:2) [45]. The results determination was performed by observation by naked eye and SYBR Green I, electrophoresis [46, 47]. At last, 8 primer sets were selected for the high flux screening platform (Table 1).

**Table 1:** The primers used for each separate LAMP reaction

Gene	Sequence (5′-3′)	Size	References
*femA*			6
F3	ATGCTGGTGGTACATCAA	18	
B3	TGGTTTAATAAAGTCACCAACAT	23	
FIP	GGTCAATGCCATGATTTAATGCATAGCATTCCGTCATTTTGCC	43	
BIP	CAGAAGATGCTGAAGATGCTGGTCAATAATTTCAGCATTGTAACC	45	
LF	AATCATTTCCCATTGCACT	22	
LB	TGTAGTTAAATTCAA	15	
*mecA*			6
F3	AAGATGGCAAAGATATTCAACT	22	
B3	AGGTTCTTTTTTATCTTCGGTTA	23	
FIP	GTGGATAGCAGTACCTGAGCCTTGATGCTAAAGTTCAAAAGAGT	44	
BIP	CCTCAAACAGGTGAATTATTAGCACCTTCGTTACTCATGCCATAC	45	
LF	TAATCATTTTTCATGTTG	18	
LB	TGTAAGCACACCTTCATATGACGT	24	
*rfbE*			2
F3	AACAGTCTTGTACAAGTCCA	20	
B3	GGTGCTTTTGATATTTTTCCG	21	
FIP	CTCTCTTTCCTCTGCGGTCC-GATGTTTTTCACACTTATTGGAT	43	
BIP	TAAGGAATCACCTTGCAGATAAACT-AGTACATTGGCATCGTGT	43	
LF	CCAGAGTTAAGATTGAT	17	
LB	CGAAACAAGGCCAGTTTTTTACC	23	
*16S rRNA*			6
F3	CGTGGGGATCAAACAGGATT	20	
B3	CATGCTCCACCGCTTGTG	18	
FIP	TAGCTGCAGCACTAAGGGGC-CCACGCCGTAAACGATGAG	39	
BIP	ACGCATTAAGCACTCCGCCT-GGGTCCCCGTCAATTCCT	38	
LF	GGAAACCCCCTAACACT	17	
LB	GGGGAGTACGACCGCAAGGT	20	
*invA*			1
F3	TCAACAATGCGGGGATCTG	19	
B3	GAAGCGTA CTGGAAAGGGAA	21	
FIP	ACRCGCCATGGTATGGATTTGTGACCATCACCAATGGTCAGC	41	
BIP	ATGATGCCGGCAATAGCGTCAAGCCAGCTTTACGGTTCCT	40	
LF	TCCGCTCTGTCTACTTATACCAT	23	
LB	TGATAAACTTCATCGCACCGTCAA	25	
*oprI*			7
F3	CTGGCTGCTGTTCTGG	16	
B3	CGCTCGTTAGCCTCGT	16	
FIP	CTGCGTCTTCGGTAGCGGGGTTGCAGCAGCCACT	34	
BIP	TCAGGCTCGCGCTGACGA-AGTCTGCTGAGCTTTCTGAG	38	
LF	TCTTTGGCTTCGAGCAGACT	20	
LB	GCCTATCGCAAGGCTGACGAA	21	
*hlyA*			8
F3	GGAGGMTACGTTGCTCAA	18	
B3	AAGCTAAACCAGTGCATTC	19	
FIP	TCGCTCCAGTTTTTATGTTGAACAC-CTTGGGATGAARTAAATTATGATCC	50	
BIP	AGCAAGCTAGCTCATTTCACAT-AGCGTAAACATTAATATTTCTCGC	46	
LF	ACTTCCATTKCTTTA	15	
LB	CGTCCATCTATTTGCCAGGTAAC	24	
*tlh*			9
F3	CGCTGACAATCGCTTCTCAT	20	
B3	GTTCTTCGCTTTGGCAATGT	20	
FIP	CTGTCACCGAGTGCAACCACTTAACCACACGATCTGGAGCA	41	
BIP	GCATCACAATGGCGCTTCCCACCGTTGGAGAAGTGACCTA	40	
LF	GTTGATTTGATCTGGCTGCATTG	23	
LB	AACCCGAACAGCTGGTTCT	19	

### Bioprocessing the separate LAMP assays into high flux screening platform

**Fig. 1:**
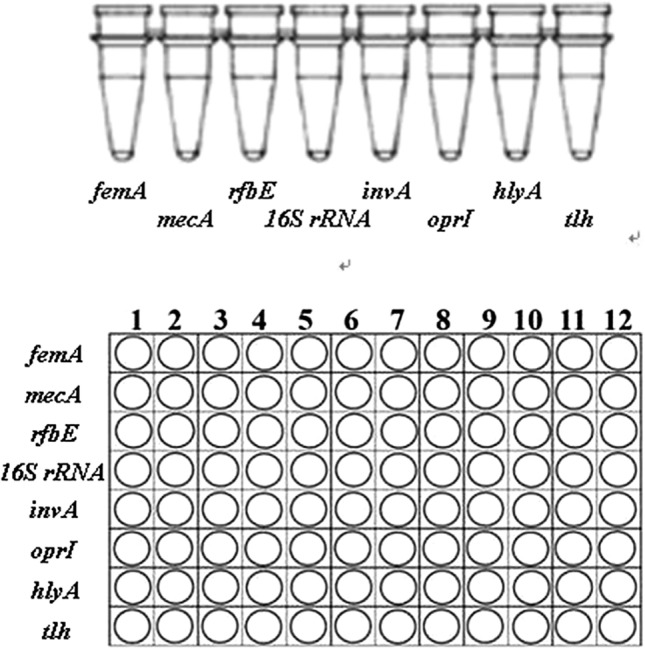
8-tube strips and PCR well plates for the biosystem platform

As mentioned above, 8 primers sets specifically targeted for rfbE, oprI, mecA, femA, 16S rRNA for staphylococci, invA, tlh and hlyA have been included [48]. As LAMP reactions were concerned, 65 °C was selected due to the efficiency, stability and reproducibility of this methodology. Also, reaction time as 45 min was selected as at this time point sufficient reaction products for results determination as well as minimal amounts of amplicons were both achieved [49–51]. For the concentration of betaine, insignificant difference was found, and thus 0.3 M was used for the consideration of minimal expense. For the ratio between calcein and Mn^2+^, 1:4 was found to be optimal and thus selected. In addition, calcein was selected to replace SYBR Green I for the color change for results determination as calcein is capable of preload in the reaction volume but SYBR Green I [52]. For application, 8 strips (PCR tubes) were employed for the detection of standard strains, and 96-well plates were employed for the detection of different samples. Different sets of primers as well as reaction volume (25 micro liters were used, with 0.3 M betaine and 1:4 of calcein and Mn^2+^) were prepared at each tube of 8 strips or 96-well plates (Fig. 1). Both 8 strips and 96-well plates could be stored under −20 °C. For standard strains detection including *E. coli*, *Pseudomonas* aeruginosa, MRSA, MSSA, MRCNS, MSCNS, *L. monocytogenes*, *Salmonella*, *V. parahaemolyticus*, 8 strips were used for 8 targets, and template DNA was loaded, followed by reaction on waterbath at 65 °C for 45 min [53–55]. Color change was determined for test results.

### Application of the high flux screening platform

In this study, a common type of food sample, cake was employed to verify the applicability of this high flux screening platform. Sample artificially contamination was performed as described previously. A total of 16 strains other than standard strains were used in the application study, including 2 *E. coli*, 2 *P. aeruginosa*, 2 MRSA, 2 MSSA, 2 MRCNS, 2 *L. monocytogenes*, 2 *Salmonella*, 2 *V. parahaemolyticus* strains. A total of four 96-well plates were used. For each 96-well plate, there are 12 panels, and for each panel, 8 detected targets were included. Experiments were performed using a double-blind method, and the demonstration is as follows [56–58]. Firstly, 2 separate operators (Group A) randomly selected different number of strains (from 1 to 8) for each of the 12 panels and conducted DNA extraction without informing the strain selection. Two other operators (Group B) further performed the detection using the 96-well plate. After the detection, 2 groups of operators compared the strains selection and detection results. Then, the same procedure was conducted vice versa, with Group B conducted strains selection and Group A conducted detection [59]. For a single 96-well plate, a total of 12 samples were detected for 8 pathogens at one reaction. Simple and rapid DNA extraction was performed as described previously, followed by loading of 5 micro liters of template DNA (with the 96-well plates placed on ice). LAMP was proceeded on waterbath at 65 °C for 45 min [60]. Color change was determined for test results. Besides, regular PCR detection was also performed as control.

## Results

### Development of separate LAMP platform and their integration

**Fig. 2:**
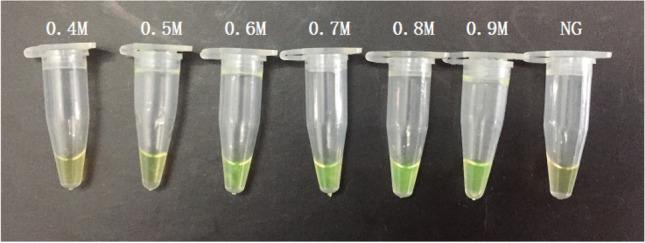
Effect of different concentration of betain on the LAMP reaction

**Fig. 3:**
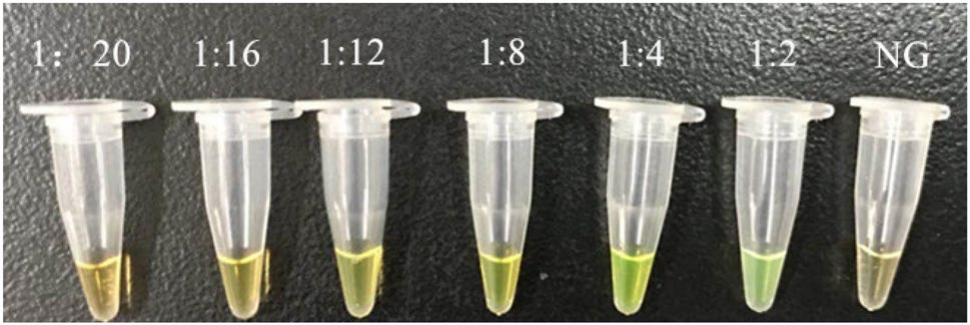
Optimization of the concentration ratio of calcein and Mn^2+^ (C(calcein) and C(Mn^2+^) is 1:20, 1:16, 1:12, 1:8, 1:4, 1:2, respectively; NG, negative control)

According to the development of LAMP assays, positive results had been obtained from DNA amplification of standard strains, with color change from orange to green by either SYBR Green I or calcein, as well as typical ladder bands pattern from electrophoresis [61–63]. Optimization of LAMP reaction was also performed, in details as follows. For reaction processed under different temperatures ranging from 59 °C to 66 °C, insignificant difference was observed between 63 °C and 65 °C, and 65 °C was selected due to the efficiency, stability (according to previous studies), applicability (significantly higher number of reported LAMP reactions had used this temperature) and reproducibility of this methodology. For reaction time ranging from 0, 15 min, 30 min, 45 min, 60 min, 75 min, 90 min, the first time point for positive results to occur was found to be 30 min, and sufficient amplicons were obtained since 45 min. As a consequence, 45 min was selected. For concentrations of betaine (0.3 M, 0.4 M, 0.5 M, 0.6 M and 0.7 M), insignificant difference was found, and thus 0.3 M was used for the consideration of minimal expense (Fig. 2). In this study, calcein was selected to replace SYBR Green I for the color change for results determination as calcein is capable of preload in the reaction volume but SYBR Green I [64, 65]. For different ratios of calcein and Mn2 + (1:20, 1:16, 1:12, 1:8, 1:4, 1:2), 1:4 was found to be optimal and thus selected (Fig. 3).

### Verification of the high flux screening platform

**Fig. 4:**
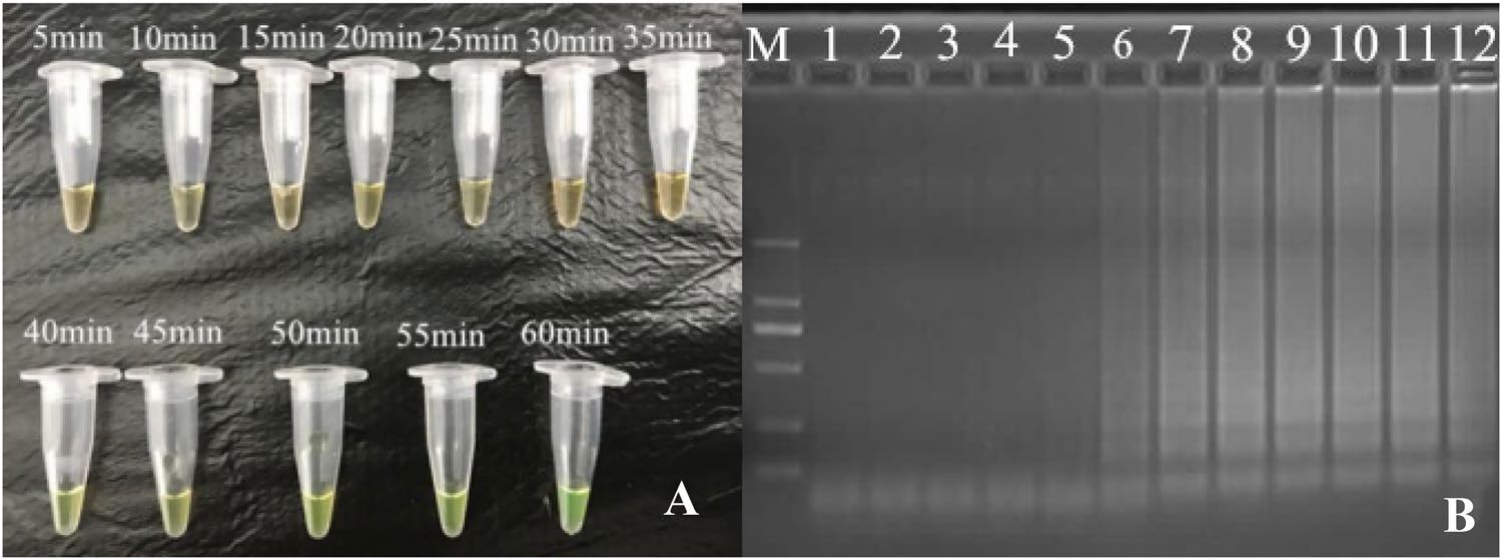
**a** Visual detection by calcein with fluorescence in LAMP amplified product, from 40 min onwards to 60 min and orange color in 5-35 min. **b** Agarose gel electrophoresis of LAMP amplification products at different time interval (5–60 min), Lane M: 2000 bp ladder marker, 1–12 are 5 min, 10 min, 15 min, 20 min, 25 min, 30 min, 35 min, 40 min, 45 min, 50 min, 55 min, 60 min)

For verification of the high flux screening platform on standard strains including *E. coli*, *Pseudomonas* aeruginosa, MRSA, MSSA, MRCNS, MSCNS, *L. monocytogenes*, *Salmonella*, *V. parahaemolyticus*, 8 strips (PCR tubes) were employed with different sets of primers, reaction volume (25 micro liters were used, with 0.3 M betaine and 1:4 of calcein and Mn2 +) preloaded at each tube of 8 strips. After loading of template DNA for each standard strain, reaction was proceeded in waterbath at 65 °C for 45 min [66]. As shown, color change from orange to green was observed for positive results, and 100% of specificity was obtained in this study (Fig. 4a). From template DNA loading to results determination, 50 min was required (Fig. 4b).

### Application of the high flux screening platform

**Fig. 5:**
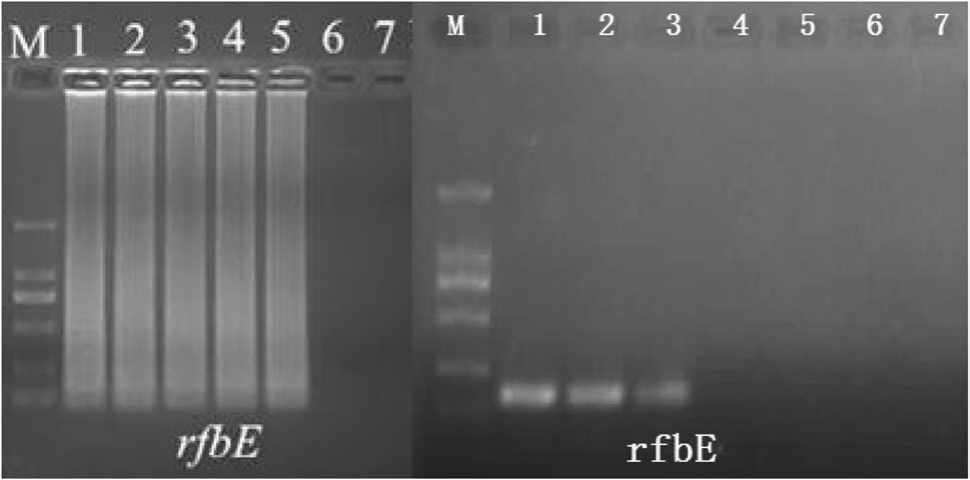
Example of electrophoresis of rfbE

According to the results, for the first round as Group A for strains selection and Group B for detection, Operator 1 had selected 1 *E. coli*, 1 *P. aeruginosa*, 2 MRSA, 1 MSSA, 1 MRCNS, 2 *L. monocytogenes*, 2 *Salmonella*, 2 *V. parahaemolyticus* strains. As shown by the detection, all of the selected strains had been diagnosed by Operator 1 from Group B. For Operator 2, 2 *E. coli*, 1 *P. aeruginosa*, 2 MRSA, 2 MSSA, 2 MRCNS, 1 *L. monocytogenes*, 1 *Salmonella*, 1 *V. parahaemolyticus* strains were selected, and all strains were correctly detected by Operator 2 from Group B [67–69]. For the second round as Group B for strains selection and Group A for detection, Operator 1 had selected 2 *E. coli*, 2 *P. aeruginosa*, 2 MRSA, 2 MSSA, 2 MRCNS, 2 *L. monocytogenes*, 0 *Salmonella*, 0 *V. parahaemolyticus* strains. As shown by the detection, all of the selected strains had been diagnosed by Operator 1 from Group A. For Operator 2, 1 *E. coli*, 1 *P. aeruginosa*, 1 MRSA, 1 MSSA, 2 MRCNS, 2 *L. monocytogenes*, 2 *Salmonella*, 2 *V. parahaemolyticus* strains were selected, and all strains were correctly detected by Operator 2 from Group A [70]. In summary, 100% of accuracy was obtained by both groups for 2 rounds of experiments (Fig. 5). As rapidity was concerned, 10–15, 8–10, 45 and 3–5 min are required for DNA extraction, template DNA loading, LAMP reaction and results determination, respectively [71, 72]. In summary, the total time consumption is 66–75 min, for simultaneous detection of 12 samples for 8 different pathogens.

## Discussion

In this study, a high flux screening based on nucleic acid isothermal amplification technique has been developed. Firstly, 8 common pathogens were selected and 8 species-specific targets had been selected and analyzed for their unique specificity. Then, 8 different sets of primers for LAMP reaction had been further designed and optimized to obtain unique reaction temperature and time. Furtherly, the 8 detection assays had been integrated into a biosystem panel for isothermal detection, including 8 strips (PCR tubes) and 96-well plates, with 8 standard strains verified for the accuracy [73–76]. At last, application of the established high flux screening platform was used for detection for 48 samples in 4 different 96-well plates, with 2 groups of 2 operators using double-blind procedure. The accuracy of 100% was obtained, with the total time consumption as 66–75 min (for 12 samples detection on 8 different pathogens) [77–79]. As concluded, through the bioprocess of the systematic platform based on LAMP technique, it’s been demonstrated to be capable of simultaneous detection of 8 pathogens, with high sensitivity, specificity, rapidity and convenience.
